# Orbital Cavernous Hemangioma Presenting with a Dome-Shaped Maculopathy-Like Appearance on Swept-Source Optical Tomography Imaging

**DOI:** 10.1155/2020/5354609

**Published:** 2020-03-25

**Authors:** Şükran Bekdemir, Ahmet Kaan Gündüz, Cevriye Cansız Ersöz

**Affiliations:** ^1^Ophthalmology Clinic, Polatlı Duatepe State Hospital, Ankara, Turkey; ^2^Department of Ophthalmology, Ankara University Faculty of Medicine, Ankara, Turkey; ^3^Department of Pathology, Ankara University Faculty of Medicine, Ankara, Turkey

## Abstract

A 43-year-old patient presented with painless proptosis, limited upgaze, and vision loss in the right eye. Funduscopic examination revealed right optic disc edema and subtle macular compression. Swept-source optical coherence tomography (SS-OCT) revealed a smooth contoured elevation of the posterior pole without any distortion of retinal structures, an appearance closely simulating dome-shaped maculopathy. Swept-source optical coherence tomography angiography (SS-OCTA) revealed normal retinal and choroidal vasculature. Orbital magnetic resonance imaging demonstrated a well-circumscribed intraconal mass compressing the globe and optic nerve in the right orbit. An anterior orbitotomy was performed, whereby the tumor was totally excised and diagnosed histopathologically as cavernous hemangioma. This case represents an orbital cavernous hemangioma touching the eyeball and producing compression of the posterior pole presenting with a dome-shaped maculopathy-like appearance on SS-OCT. SS-OCT and SS-OCTA are important noninvasive tools for evaluating the retinal and choroidal effects in orbital tumors.

## 1. Introduction

Orbital cavernous hemangioma or orbital venous malformation by the newer nomenclature is the most common benign orbital tumor in adults. It typically presents with unilateral progressive painless proptosis in middle-aged females. Other presenting features include vision loss, restricted eye movements, diplopia, choroidal folds, and optic disc edema [1, 2]. Orbital imaging with computed tomography and magnetic resonance imaging (MRI) usually demonstrates a round- or oval-shaped well-circumscribed orbital tumor and may identify features such as optic nerve or globe compression. In recent years, optical coherence tomography (OCT) and optical coherence tomography angiography (OCTA) have become important diagnostic tools in evalauating retinal and choroidal diseases.

We herein report an interesting case of orbital cavernous hemangioma touching the eyeball and producing compression of the posterior pole simulating the appearance of dome-shaped maculopathy (DSM) on swept-source optical coherence tomography (SS-OCT).

## 2. Case Presentation

**Figure 1:**
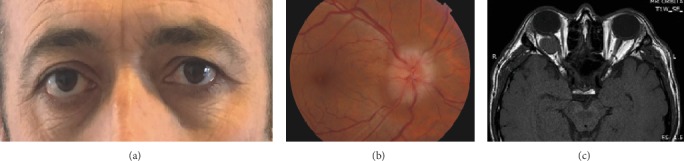
(a) Facial appearance of the patient at presentation. (b) Color fundus photograph of the right eye shows prominent optic disc edema and subtle macular compression. (c) Orbital T1-weighted axial magnetic resonance imaging shows an intraconal tumor isointense to the extraocular muscles producing compression of the globe and indenting the optic nerve.

A 43-year-old male presented with vision loss in the right eye of 2-month duration. No history of systemic or ocular disease and trauma was present. Thyroid function tests were within normal limits. There was no edema or ecchymosis on either side. Visual acuity was 20/400 OD and 20/20 OS. There was no myopia or hypermetropia in either eye. Intraocular pressures were 17 mmHg OU. There was no relative afferent pupil defect bilaterally. Eye motility was normal in both eyes with the exception of limited upgaze in the right eye. There was 7 mm proptosis on the right side (Figure 1(a)). Fundus examination revealed prominent optic disc edema, subtle macular compression, and normal retinal vessels. Choroidal folds were not present (Figure 1(b)). Anterior segment and fundus examination in the left eye were normal. The patient underwent orbital MRI, which revealed an intraconal ovoid tumor compressing the globe posteriorly. The tumor displaced the lateral rectus laterally and optic nerve superomedially. The mass was isointense with respect to the extraocular muscles on T1-weighted images, hyperintense on T2-weighted images, and demonstrated contrast enhancement (Figure 1(c)).

**Figure 2:**
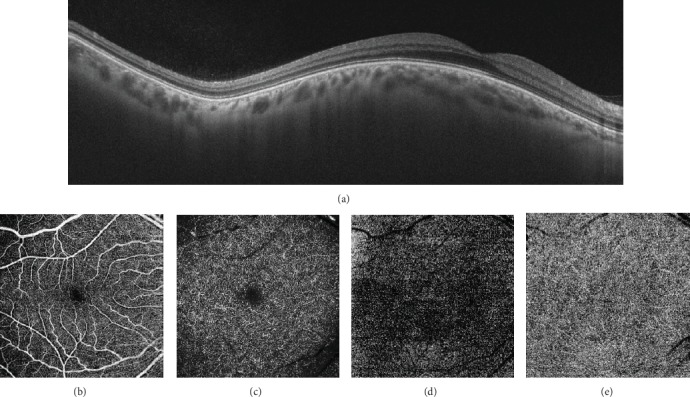
(a) Swept-source optical coherence tomography through the fovea reveals a smooth contoured elevation of the posterior pole without any distortion of retinal structures, giving a dome-shaped maculopathy-like appearance. (b-e) Swept-source optical coherence tomography angiography images of superficial retinal plexus (b), deep retinal plexus (c), outer retina (d), and choriocapillaris (e) show normal retinal and choroidal vasculature patterns.

**Figure 3:**
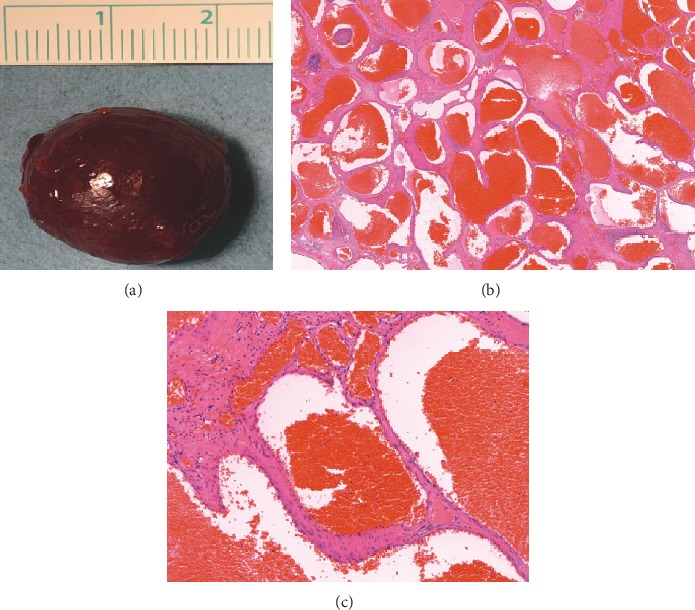
(a) Gross appearance of the excised orbital tumor showing the reddish well-circumscribed mass. (b) Histopathological examination reveals enlarged vascular channels with thick walls, the lumens of which were filled with erythrocytes, consistent with orbital cavernous hemangioma (H.E.×20). (c) Higher magnification view of vascular structures lined by endothelium and filled with erythrocytes (H.E.×100).

**Figure 4:**
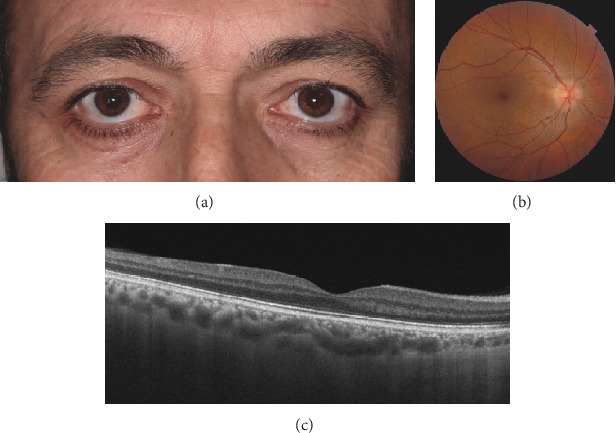
(a) Clinical appearance of the patient one month after orbitotomy showing that the right inferotemporal orbitotomy skin incision has nicely healed. (b) Fundus photograph shows significant resolution of optic disc edema and no macular compression. (c) Swept-source optical coherence tomography shows complete resolution of dome-shaped maculopathy-like appearance.

SS-OCT (DRI OCT Triton plus, Topcon, Tokyo, Japan) operating at a speed of 100.000 A-scans/second revealed a smooth contoured elevation of the posterior pole without any distortion of retinal structures, an appearance closely simulating DSM (Figures 2(a)). There was no retinal traction or choroidal folds. Subfoveal choroidal thickness as measured by the automated choroidal scleral interface segmentation was 284 *μ*m. Scleral thickness could not be measured. Swept-source optical coherence tomography angiography (SS-OCTA) revealed normal vascularisation in superficial and deep retinal plexi, outer retina, and choriocapillaris (Figure 2(b)–2(e)). An anterior orbitotomy was performed via the inferotemporal skin incision, and the tumor was totally removed with the help of a cryoprobe. The base diameters of the excised reddish, smooth edged, capsulated mass measured approximately 2.5 × 1.5 cm (Figure 3(a)). Histopathological examination revealed enlarged vascular channels with thick walls, the lumens of which were filled with erythrocytes. The histopathological diagnosis was rendered as orbital cavernous hemangioma (Figures 3(b) and 3(c)). Ophthalmological examination 1 month after the surgery showed that the right orbitotomy skin incision had nicely healed (Figure 4(a)). Visual acuity improved to 20/20, and optic disc edema resolved significantly in the right eye (Figure 4(b)). SS-OCT showed complete resolution of the DSM-like appearance at the posterior pole (Figure 4(c)).

## 3. Discussion

Optical coherence tomography is a method that provides high-resolution cross-sectional imaging in biological tissues. In ophthalmology, it is widely used for noninvasive imaging of the posterior pole including the optic disc, macula, and choroid, in addition to anterior segment structures [3]. In our case, there was elevation in the macular area due to the orbital mass which compressed the globe from the posterior aspect. SS-OCT revealed the presence of a smooth contoured elevation of the posterior pole, an appearance closely simulating DSM. There was no distortion of retinal structures on SS-OCT and no retinal and choroidal vasculature changes were noted on SS-OCTA.

Dome-shaped maculopathy was first described as anterior protrusion of macula in the posterior staphyloma area of highly myopic patients. Subsequent studies indicated that DSM may also be present without posterior staphyloma [4, 5]. Although the pathophysiology is not clear, mechanisms such as localized thickening of the choroid, vitreoretinal traction, and posterior eye-wall collapse have been suggested as responsible factors. Subsequently, focal thickening of the subfoveal sclera has been proposed as a more likely mechanism, though the reason for the thickening is unknown [6]. Dome-shaped maculopathy is associated with high myopia (>6 diopters and axial length >26.5 mm) and its presence is positively correlated with the severity of myopic maculopathy. The prevalence of DSM in the general population is unknown. However, unilateral or bilateral DSM occurs in 10-20% of highly myopic patients [7–10]. Dome-shaped maculopathy was subsequently reported to occur in hereditary retinal dystrophies, central serous chorioretinopathy, and also in hypermetropic and emmetropic patients [5]. Dome-shaped maculopathy can be complicated by serous retinal detachment, choroidal neovascularization, and macular retinal pigment epithelial atrophy [8]. Our case was not myopic or hypermetropic in either eye. In addition, there were no evidence of other retinal problems alluded to in previous studies as possible etiologic factors for DSM. Choroidal thickness measurement was normal.

This case presents an interesting example of a DSM-like appearance on SS-OCT diagnosed in a patient with globe compression by an orbital cavernous hemangioma. Indirect ophthalmoscopy, fluorescein angiography, B mode ultrasonography, and MRI may be helpful in identifying globe compression caused by orbital tumors. Optical coherence tomography and OCTA, as noninvasive diagnostic tools, may reveal the retinal and choroidal changes better in such circumstances and without injection of dye.
